# The Automaton with Articulated Voice

**Published:** 1844-04

**Authors:** 


*The Automaton with Articulated Voice.*—We have had the pleasure of examining privately this *chef-d'œuvre* of human ingenuity—the result of eighteen years' unceasing labor, by a German, named Faber. It is constructed upon the model of the human organs of voice, the tongue, larynx, &c., being made of caoutchouc. As voice is the sound produced by air driven from the lungs through the larynx, causing a vibration of the chordæ vocales, it is a function of *animal* life; but this function, in animals inferior to man, as well as in the idiot, is limited to the production of the *simple* or *instinctive* voice; while, in intellectual man, it becomes sufficiently complicated for the purpose of articulation. This is regarded as an evidence of man's intellectual superiority. Here, however, we find the same phenomena produced by an apparatus of caoutchouc and a bellows!

The automaton is represented by a bearded Turk, and the articulations are produced by playing upon sixteen keys. We were quite surprised at being addressed by the automaton, in words very distinctly articulated, thus: "Wel-come Doc-tor For-ry. Please excuse my slow e-nun-ci-a-tion." After giving various other illustrations of his vocal powers, the automaton sang "Hail Columbia," &c.; as we were about leaving, he said, "Gen-tle-men, I thank you for your vis-it."

But, after all, *cui est bono?—New York Jour. Med.*

